# Sagnac interferometry for high-sensitivity optical measurements of spin-orbit torque

**DOI:** 10.1126/sciadv.adi9039

**Published:** 2023-09-08

**Authors:** Saba Karimeddiny, Thow Min Jerald Cham, Orion Smedley, Daniel C. Ralph, Yunqiu Kelly Luo

**Affiliations:** ^1^ Cornell University, Ithaca, NY 14850, USA.; ^2^ Cornell Kavli Institute at Cornell, Ithaca, NY 14853, USA.; ^3^ Department of Physics and Astronomy, University of Southern California, Los Angeles, CA 90089, USA.

## Abstract

Sagnac interferometry can provide a substantial improvement in signal-to-noise ratio compared to conventional magnetic imaging based on the magneto-optical Kerr effect. We show that this improvement is sufficient to allow quantitative measurements of current-induced magnetic deflections due to spin-orbit torque even in thin-film magnetic samples with perpendicular magnetic anisotropy, for which the Kerr rotation is second order in the magnetic deflection. Sagnac interferometry can also be applied beneficially for samples with in-plane anisotropy, for which the Kerr rotation is first order in the deflection angle. Optical measurements based on Sagnac interferometry can therefore provide a cross-check on electrical techniques for measuring spin-orbit torque. Different electrical techniques commonly give quantitatively inconsistent results so that Sagnac interferometry can help to identify which techniques are affected by unidentified artifacts.

## Teaser

Sagnac interferometry provides high-sensitivity optical readout of spin-orbit torques for efficient manipulation of nanomagnets.

## INTRODUCTION

Spin-orbit torques (SOTs) (*1*, *2*) are of interest for achieving efficient manipulation of magnetization for low-power nonvolatile magnetic memory technologies. SOTs are produced when a charge current is applied through a channel with strong spin-orbit coupling, giving rise to a transverse spin current. This spin current can exert a spin-transfer torque on an adjacent ferromagnet (FM), allowing for low-power electrical control of its magnetic orientation. Accurate quantitative measurements of the efficiency of SOTs are important for understanding the microscopic mechanisms of the torque and for optimizing materials for applications. The work-horse techniques for this purpose have been electrical measurements of current-induced magnetic reorientation with readout based on the magnetoresistance properties of the samples (*2*–*19*), but these have some shortcomings. One must be careful to separate thermoelectric voltages from the torque signals (*20*, *21*), and, even when performed carefully, different electrical techniques can often produce quantitatively inconsistent measurements, indicating that some may be affected by artifacts that are not yet understood (*22*–*26*). Furthermore, in cases when one wishes to measure SOTs acting on insulating magnetic layers, electrical measurements provide much lower signal levels compared to metallic magnets due to decreased magnetoresistance. Optical techniques based on the magneto-optical Kerr effect (MOKE) have been introduced as an alternative to quantify SOTs (*27*–*29*), but, in previous studies, the sensitivity of MOKE measurements has been insufficient to measure current-induced small-angle magnetic deflection in samples with perpendicular magnetic anisotropy (PMA)—the most-direct approach for quantifying the torque in the class of samples of primary interest for high-density memory applications.

In this work, we demonstrate improved optical detection of SOTs by using a fiber Sagnac interferometer to measure current-induced small-angle magnetic tilting. Unlike conventional MOKE measurements that rely on a single-laser beam, Sagnac interferometry uses the modulated phase difference of two coherent beams that travel along overlapping paths and are incident on the sample with opposite helicities. By detecting the resulting light intensity of the interfering beams, we achieve signal-to-noise ratios at least 50 to 100 times greater than conventional MOKE performed on a PMA metallic thin film (section S5). This allows us to perform accurate, highly sensitive measurements of the spin-orbit-torque vectors in both PMA samples and in-plane anisotropy samples, based on direct optical detection of magnetization deflection in the out-of-plane (OOP) direction.

## RESULTS

### Principles of Sagnac interferometry

**Fig. 1.**
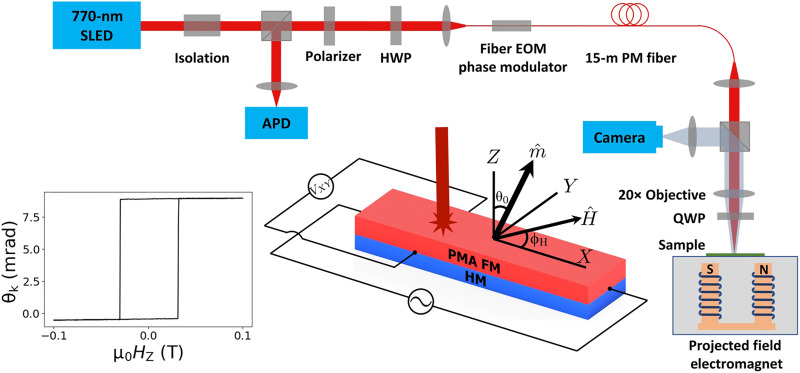
Schematic of the Sagnac interferometer. The left inset shows the Sagnac signal for OOP magnetic field–swept hysteresis of a Pt (4 nm)/Co (1.15 nm)/MgO device with OOP anisotropy μ_0_*M*_eff_ ≍ − 0.42 T; this is the same device for which we show data in Fig. 2. The middle inset depicts the device structure and coordinate definitions. SLED, superluminescent diode.

Our Sagnac interferometer consists of free-space optics and a 15-m-long single-mode polarization-maintaining (PM) fiber in a compact table-top setup. As shown in Fig. 1, two spatially overlapping, orthogonal linearly polarized beams travel inside the fiber along its fast and slow axes. Both beams pass through a quarter-wave plate (QWP) to become left and right circularly polarized, reflect from the sample, and then pass back through the QWP to reenter the fiber, thereby returning via the opposite fiber axis. The two beams therefore traverse the same optical path (in opposite directions) with phase and amplitude differences determined by the differences in reflection of left and right circularly polarized light from the sample. To measure this phase difference (i.e., 2θ_k_, where θ_k_ is the Kerr rotation angle of the sample), one can modulate the phase difference of the two beams using an electro-optic modulator (EOM). When the EOM phase modulation frequency ω matches the total optical path τ [ω = π*/*τ = 2π (3.347 MHz) for our apparatus], the Kerr rotation can be quantified as

$$\theta_{k}=-\frac{1}{2}\;\arctan\left[\frac{V_{\mathrm{APD}}^{1\omega }\;J_{2}(2\phi_{m})}{V_{\mathrm{APD}}^{2\omega }\;J1(2\phi_{m})}\right]$$ (1)

where $V_{\mathrm{APD}}^{1\omega }$ and $V_{\mathrm{APD}}^{2\omega }$ are the first and second harmonic intensity signals from the interferometer, ϕ_m_ is the EOM phase modulation depth between the fast and slow axes, and *J*_1(2)_ are the Bessel functions. Details of this derivation and more information about the Sagnac apparatus and its operation are provided in the Supplementary Materials.

For demonstration purposes, we will describe measurements on two thickness series of Pt (4 nm)/Co (0.86 to 1.24 nm)/MgO (1.9 nm)/Ta (2 nm) and Pt (4 nm)/Co (1.39 to 2.08 nm)/MgO (1.9 nm)/Ta (2 nm) samples in which the Co layer is deposited as a wedge to provide a range of thicknesses on the same wafer. The samples are made by sputtering on a high-resistivity Si/SiO_2_ wafer with a 1.5-nm Ta seed layer. They are patterned into 20 μm–by–80 μm Hall bars with 6-μm side contacts by photolithography and ion milling. The Pt resistivities for each series are 40 and 54 μohms•cm, respectively (see section S6B for details). All measurements are performed at room temperature.

Magnetic hysteresis loops can be obtained by measuring θ_k_ while sweeping an external magnetic field. The lower-left inset in Fig. 1 shows a hysteresis loop as a function of OOP magnetic field for a Pt (4 nm)/Co (1.15 nm)/MgO bilayer sample with PMA. We achieve a sensitivity in measuring θ_k_ of better than $5\;\mu \mathrm{rad}/\sqrt{\mathrm{Hz}}$ for an average laser power of 1 μW at the avalanche photodetector (APD; Fig. 1), sufficient so that the noise level is not easily visible in Fig. 1. While conventional MOKE can achieve comparable sensitivity using external modulation of magnetic field, electric field, or current (*30*, *31*), these methods are not applicable for measuring hysteresis curves of FMs.

The Sagnac signal is sensitive only to the OOP component *m_z_* of the magnetization unit vector, with no measurable dependence on the in-plane components. For linearly polarized light incident on the sample in the normal direction, the quadratic MOKE effect does allow a second-order dependence on the in-plane magnetization components in that the total Kerr rotation can have the form (*28*)

$$\theta_{k}=\kappa m_{z}+\beta_{Q}m_{x}m_{y}$$ (2)

where κ is a material-specific constant of proportionality relating the OOP net magnetization to θ_k_, β_Q_ is the quadratic MOKE coupling parameter, and *m*_x_ and *m*_y_ are defined such that *x* lies along the plane of light polarization. However, we calculate that the contribution of quadratic MOKE to the Sagnac signal is approximately a factor of 10^−5^ smaller than the κ*m*_z_ contribution (see section S3). Furthermore, the quadratic MOKE contribution to the Sagnac signal should introduce a dependence ∝ sin(2ϕ), where ϕ is the angle between the in-plane magnetization and a reference plane of light polarization. No such dependence is measurable in Sagnac measurements if we apply in-plane field of fixed magnitude and then rotate ϕ (see fig. S2). On the basis of both calculations and measurements, we therefore conclude that the Sagnac signal depends measurably only on *m*_z_. The absence of dependence on the in-plane magnetization components simplifies the Sagnac measurements of SOT relative to, e.g., electrical measurements of the second harmonic Hall effect (*6*), for which planar Hall signals are assumed to affect the signals in addition to the anomalous Hall effect.

### Using Sagnac interferometry to measure SOTs

We measure current-induced torques by applying a calibrated low-frequency ac along the *X* direction (ω_e_ = 3.27 kHz) to the heavy metal/FM bilayers and measuring the resulting small-angle deflection of the magnetization. The deflection is detected from the Sagnac signal using a side-band demodulation technique, allowing us to simultaneously measure both the steady-state value θ_k_ demodulated at the EOM frequency ω and the current-induced change ∆θ_k_ at the lower side-band frequency ω − ω_e_. We achieve a current-modulated Kerr rotation sensitivity of $3\;\mu \mathrm{rad}/\sqrt{\mathrm{Hz}}$, allowing us to detect small changes of *m*_z_ due to current-induced torques. The ac frequency ω_e_ is sufficiently low for the magnetic dynamics to be quasi steady state. Therefore, by balancing torques within the Landau-Lifshitz-Gilbert-Slonczewski equation (*32*) in steady state, the current-induced damping-like and field-like effective torques (per unit magnetization) $\tau_{\mathrm{DL}}^{0}$ and $\tau_{\mathrm{FL}}^{0}$ can be determined from the deflection of the magnetic unit vector $\text{Δ}\hat{m}$ according to

$${\gamma \mu }_{0}\text{Δ}\hat{m}\times {\vec{H}}_{\mathrm{eff}}=\tau_{\mathrm{DL}}^{0}\;\hat{m}\times (\hat{\sigma }\times \hat{m})+\tau_{\mathrm{FL}}^{0}\hat{\sigma }\times \hat{m}$$ (3)

where γ = 2μ_B_/ħ is the gyromagnetic ratio with μ_B_ the Bohr magneton and ${\vec{H}}_{\mathrm{eff}}$ is the vector sum of the anisotropy field and any applied magnetic field. We assume here that the spin-source layer has high symmetry so that the orientation of the current-induced spin polarization is parallel to the *Y* direction, i.e., in the sample plane and perpendicular to the charge current (shown in Fig. 1, middle inset).

### Samples with PMA

We first consider the case of samples with PMA, which is the more difficult case for optical measurements of SOT because the measured changes in the OOP magnetization are second order in small-angle tilting from the OOP direction. In the presence of an in-plane applied magnetic field *H* and in the absence of applied current, the equilibrium polar angle of the magnetization θ_0_ (measured from the *z* axis) satisfies sin θ_0_ = *H/*|*M*_eff_|, where the effective magnetization μ_0_*M*_eff_ = μ_0_*M*_s_ − 2 *K*_⊥_*/M*_s_ is the saturation magnetization minus the OOP anisotropy (with μ_0_*M*_eff_ negative for PMA samples) (*6*). Therefore, Kerr rotation associated with the magnetic field–induced equilibrium tilt angle (θ_0_) is approximately

$$\theta_{k}=\pm \kappa \left(1-\frac{H^{2}}{2M_{\mathrm{eff}}^{2}}\right)$$ (4)

where the ± corresponds to the initial OOP magnetization *m*_*z*_ = ±1 (see section S4 for details). From Eq. 3, the current-driven effective field in the *X* direction corresponds to the damping-like torque: $\mu_{0}\text{Δ}H_{X}=\mp \tau_{\mathrm{DL}}^{0}/\gamma$. The current-induced effective field in the *Y* direction is the sum of the field-like spin-orbit-torque contribution and the Ørsted field $\mu_{0}\text{Δ}H_{Y}=\mu_{0}H_{\mathrm{Oe}}+\tau_{\mathrm{FL}}^{0}/\gamma$

**Fig. 2.**
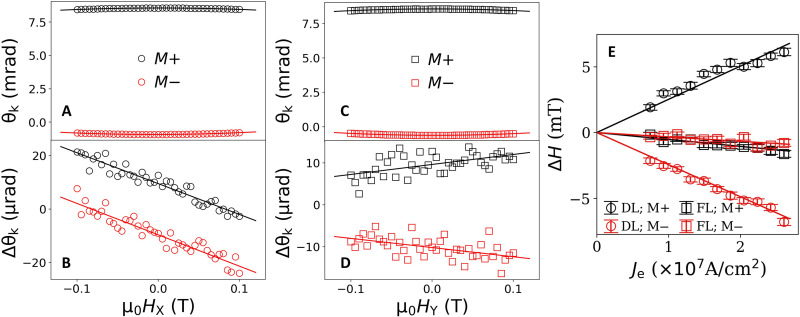
Sagnac interferometry measurements of current-induced torque for a Pt (4 nm)/Co (1.15 nm)/MgO sample with PMA. (**A** and **B**) The Sagnac signals θ_k_ and ∆θ_k_ for an in-plane magnetic field applied in the *X* direction, for which ∆θ_k_ provides a measurement of the damping-like torque. (**C** and **D**) Corresponding signals for an in-plane magnetic field applied in the *Y* direction, for which ∆θ_k_ provides a measurement of the field-like torque. (**E**) Current-induced effective fields as a function of current density in the Pt layer, with linear fits to extract the spin-torque efficiencies.

To measure the current-driven effective fields ∆*H*_X_ and ∆*H*_Y_ for samples with PMA, we apply an in-plane magnetic field along the *X* or *Y* axis (*H*_X_ at ϕ_H_ = 0 or *H*_Y_ at ϕ_H_ = π*/*2, where ϕ_H_ is the angle of the in-plane field relative to the current direction) for both of the cases *m*_z_ = ±1 and perform simultaneous measurements of θ_k_ and ∆θ_k_. The left two panels of Fig. 2 show the results for the same PMA Pt (4 nm)/Co (1.15 nm)/MgO bilayer, for which the OOP hysteresis curve is shown in Fig. 1, for an ac amplitude of 15 mA corresponding to a current density in the Pt layer of 1.9 × 10^7^ A/cm^2^. Because ∆*H*_X_ and ∆*H*_Y_ cause small oscillations of the magnetization, the current-induced Kerr rotation (derived in section S4) can be approximated as

$$\text{Δ}\theta_{k}=\mp \kappa (\text{Δ}H_{X}\;\cos\phi_{H}+\text{Δ}H_{Y}\;\sin\phi_{H})\frac{H}{M_{\mathrm{eff}}^{2}}$$ (5)

Therefore, ∆*H_X_* and ∆*H_Y_* can be extracted on the basis of Eqs. 4 and 5 as

$$\text{Δ}H_{X}=\frac{d\text{Δ}\theta_{k}(\phi_{H}=0)}{dH}{\left(\frac{d^{2}\theta_{k}}{dH^{2}}\right)}^{-1}$$ (6)

$$\text{Δ}H_{Y}=\frac{d\text{Δ}\theta_{k}(\phi_{H}=\pi /2)}{dH}\;{\left(\frac{d^{2}\theta_{k}}{dH^{2}}\right)}^{-1}$$ (7)

For the current amplitude of 15 mA, we find μ_0_∆*H*_X_ = μ_0_∆*H*_DL_ = 5.0(3) mT and μ_0_∆*H*_Y_ = μ_0_∆*H*_FL_ = −0.9(2) mT for *m_z_* = +1, and μ_0_∆*H*_X_ = −μ_0_∆*H*_DL_ = −5.1(3) mT and μ_0_∆*H*_Y_ = μ_0_∆*H*_FL_ = −0.9(2) mT for *m*_z_ = −1. These signs are consistent with the directions of the damping-like and field-like effective fields measured by harmonic Hall and spin-torque ferromagnetic resonance (ST-FMR) from Pt (*4*, *5*, *33*).

We can also express these results in terms of dimensionless SOT efficiencies ξ_DL_ and ξ_FL_

$$\xi_{\mathrm{DL}(\mathrm{FL})}=\tau_{\mathrm{DL}(\mathrm{FL})}^{0}\frac{eM_{s}t_{\mathrm{Co}}}{\mu_{B}J_{e}}$$ (8)

where *J*_e_ is the electric current density in the spin source layer, *M*_s_ is the saturation magnetization of the FM, and *t*_Co_ is the thickness of the FM cobalt layer. (Note by this definition that ξ_FL_ contains contributions from both the Ørsted torque and the field-like SOT.) For each of our samples, we calibrate the saturation magnetization per unit area *M*_s_*t*_Co_ using vibrating-sample magnetometry (VSM) on 3 mm–by–3 mm thin films diced from the wafer adjacent to the patterned devices (see section S6C). We calculate *J*_e_ using a parallel-conduction model after determining the thickness-dependent conductivities of the different layers in the heterostructure (see section S6B). For the most accurate determination of the torque efficiencies, we measure ∆*H*_X_ and ∆*H*_Y_ for a sequence of applied voltage amplitudes for *m*_z_ = +1 and fit to a linear dependence (Fig. 2E). We can then extract ξ_DL(FL)_ based on the fitted linear slope from Eq. 8. For the PMA Pt (4 nm)/Co (1.15 nm)/MgO bilayer, we find ξ_DL_ = 0.132(2) and ξ_FL_ = −0.023(2). We will analyze below the results for full thickness series of the Co layer.

### Samples with in-plane magnetic anisotropy

For the case of samples with in-plane anisotropy, the current-induced changes in *m*_z_ are first order in the tilting angle for OOP magnetic deflections. On the basis of Eq. 3, for in-plane magnetization, the damping-like torque corresponds to an OOP effective field, while the field-like torque gives an in-plane effective field. Therefore, our Sagnac MOKE interferometry measures only the OOP magnetic deflection from the damping-like effective field, with the maximum magnitude (for ϕ_H_ = 0) of μ_0_Δ*H*_DL_ = $\tau_{\mathrm{DL}}^{0}$/γ, and ∆θ_k_ (derived in section S4) can be expressed as

$$\text{Δ}\theta_{k}=\frac{\kappa \text{Δ}H_{\mathrm{DL}}\cos\phi_{H}}{H+M_{\mathrm{eff}}}$$ (9)

**Fig. 3.**
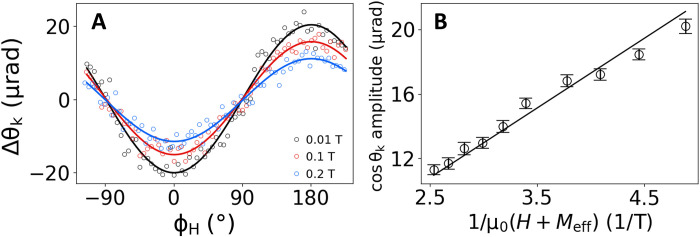
Sagnac interferometry measurements of current-induced torque for a Pt (4 nm)/Co (1.42 nm)/MgO sample with in-plane magnetic anisotropy. (**A**) ∆θ_k_ as a function of in-plane magnetic field angle ϕ_H_ at 0.1, 0.15, and 0.2 T. (**B**) Amplitudes of the cos ϕ_H_ component for different applied field magnitudes. The linear slope as a function of 1*/*μ_0_(*H* + *M*_eff_) allows extraction of the damping-like effective field based on Eq. 9.

Figure 3A shows ∆θ_k_ as a function of the angle of the in-plane magnetic field ϕ_H_ with constant magnitudes of magnetic field (μ_0_*H* = 0.1, 0.15, and 0.2 T) and a current amplitude of 8 mA for a bilayer with the composition Pt (4 nm)/Co (1.42 nm)/MgO, which has in-plane magnetic anisotropy. To quantify ∆*H*_DL_, we fit the amplitude of the cosϕ_H_ components as a function of 1*/*[μ_0_(*H* + *M*_eff_)] and perform a linear fit as shown in Fig. 3B. We also determine the effective magnetization *M*_eff_ for each device from ST-FMR (section S6D). For the device featured in Fig. 3, μ_0_*M*_eff_ = 0.195 T, and the final result of the measurement is μ_0_∆*H*_DL_ = 3.0(1) mT, corresponding to ξ_DL_ = 0.10(1).

### Results for samples over the full-thickness range

**Fig. 4.**
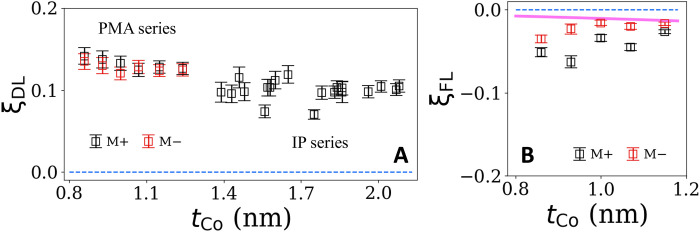
Final results for the damping-like and field-like spin-orbit-torque efficiencies for the sample series substrate/Ta (1.5)/Pt (4)/Co (0.85 to 2.1)/MgO (1.9)/Ta (2). The numbers in parentheses are thicknesses in nanometers. The pink line in (**B**) indicates the estimated Oersted torque based on the calculated current density. The larger error bars for the in-plane (IP) series compared to the PMA series in (**A**) are primarily a result of greater sample-to-sample scatter in the VSM measurements of *M*_s_*t*_Co_ rather than uncertainty in the Sagnac measurement.

The results of the Sagnac-interferometer measurements of SOT efficiencies for the full range of thicknesses for the Pt (4 nm)/Co (0.85 to 2.1 nm)/MgO are shown in Fig. 4. By varying the Co thickness, competition between the in-plane shape anisotropy and interface PMA gives rise to different values of *M*_eff_ (plotted in fig. S8). We observe at most only a weak dependence of ξ_DL_ on the Co layer thickness (Fig. 4, A and B). This is expected as long as the Co layer is sufficiently thick for full absorption of the transverse component of the incoming spin current and qualitatively consistent with previous electrical measurements (*34*). The values of ξ_DL_ obtained by the Sagnac measurements on PMA and in-plane samples are consistent, which is often not the case for electrically based second harmonic Hall measurements of SOT (*35*). This value that we find for the damping-like SOT efficiency is also in quantitative agreement with ST-FMR measurements with similar Pt resistivity (*22*, *36*, *37*). Because the Sagnac interferometry is sensitive only to OOP magnetic deflections, we obtain measurements of the current-induced field-like torque only for the PMA samples, in which case the field-like torque efficiency ξ_FL_ is considerably smaller than ξ_DL_ as shown in Fig. 4B. The estimated Oersted torque is of similar amplitude as indicated in pink line in Fig. 4B. This indicates that the field-like SOT is at most a small contribution.

## DISCUSSION

We have shown that Sagnac interferometry provides a sufficient improvement in the signal-to-noise ratio compared to conventional MOKE to enable for the first time optical measurements of SOT efficiencies even for thin-film magnetic samples with OOP magnetic anisotropy for which the Kerr signal is second order in the magnetic deflection angle. The Sagnac technique also allows optical measurements of the damping-like component of SOT for samples with in-plane magnetic anisotropy, the component of torque that causes OOP magnetic deflections in this geometry. [Measurements for the in-plane geometry have also been performed previously using conventional MOKE (*27*–*29*).] Optical measurements provide the capability to perform quantitative studies of SOT in samples for which magnetoresistance signals are small (e.g., insulating magnetic layers). They can also provide an important cross-check on electrical measurements of SOT, to identify cases in which the electrical measurements are affected by unknown artifacts. In our Pt/Co wedge series samples, we find that the Sagnac measurements of the damping-like SOT efficiency are in reasonable quantitative agreement throughout the thickness series for the magnetic layer, for samples with both PMA and in-plane anisotropy. These values are also in good agreement with ST-FRM measurements with similar Pt resistivity (*22*, *36*, *37*). However, as we have noted in a separate arXiv posting, low-frequency second harmonic electrical measurements for the PMA samples yield results that are inconsistent with both the Sagnac measurements and the ST-FMR results on the in-plane samples. The Sagnac results therefore provide confirmation of the ST-FMR values and reason to question the accuracy of the second harmonic electrical technique applied to PMA samples (at least for PMA samples in which the planar Hall effect is substantial) (*35*).

## MATERIALS AND METHODS

### Sample fabrication

The sample heterostructures are grown by dc magnetron sputtering at a base pressure of less than 3 × 10^−8^ torr on high-resistivity, surface-passivated Si/SiO_2_ substrates. Hall bars are patterned using photolithography and ion mill etching, and, then, Ti/Pt contacts are deposited using photolithography, sputter deposition, and liftoff. The Co is deposited with a continuous thickness gradient (“wedge”) across the 100 mm wafers, and all devices measured have their current flow direction oriented along the thickness gradient. The Hall bar devices measured are 20 μm by 80 μm in size, and the change in Co thickness is negligible on this scale, i.e., the gradient over 80 μm is orders of magnitude smaller than the root mean square film roughness. The Ta underlayer is used to seed a smooth growth of subsequent films, and the MgO/Ta forms a cap to minimize oxidation of the Co layer.

### Sagnac interferometer design

Our Sagnac interferometer [further details can be found in (*38*)], modeled after those in (*39*, *40*), is shown in Fig. 1. The beamline begins with a 770-nm superluminescent diode. The beam goes through a pair of Faraday isolators that provide *>*65 dB of backward isolation and prevent back reflections into the diode that would cause intensity fluctuations and other source instabilities. Next, the beam goes through a beam splitter, polarizer, and half-wave plate that prepare the beam polarization to be 45° with respect to the slow axis of a single-mode PM fiber into which it is focused. The beam will henceforth be discussed as an equal combination of two separate beams of linearly polarized light: one polarized along the slow axis and one polarized along the fast axis of the PM fiber. A fiber electro-optic phase modulator (EOSPACE Inc.) applies time-dependent phase modulation to the beam traveling along the slow and fast axes with different amplitude modulation depths: ϕ_∥_ or ϕ_⊥_, respectively. The difference of these two amplitude modulation depths, ϕ_m_ = ϕ_∥_ − ϕ_⊥_, is controlled by a lock-in oscillator voltage output (Zurich Instruments, HF2LI). The beam then travels along 15 m of PM fiber, whereupon it is collimated and focused by a long–working distance objective through a QWP and onto a sample. The QWP is oriented such that one beam is converted to left circularly polarized light, and the other is converted to right circularly polarized light. The beams then reflect off of a sample, exchanging the handedness of the beams and, if the sample is magnetic, imparting both the effects of circular dichroism and circular birefringence; the latter is equivalent to a Kerr rotation of a linearly polarized light. Upon reflection, the two beams (now exchanged) backpropagate and acquire a net phase difference of ϕ_m_[sin(ω(*t* + τ) − sin(ω(*t*)] at the EOM, where τ is the time that it takes for the light to make the round trip back. The two beams interfere to produce homodyne intensity oscillations at the EOM frequency. The backpropagating beams are then routed by the beam splitter and focused into a broadband APD. The APD’s output voltage is measured by a lock-in amplifier that references the driving frequency of the EOM, ω. To simplify the interpretation of the signal, the frequency ω is tuned such that ω = π*/*τ (*39*) {[2π(3.347 MHz)] for our apparatus}. To maximize the Kerr rotation signal, the phase modulation depth ϕ_m_ is set by tuning the magnitude of ac voltage (*V*_pk_ = 0.65 V) applied to the EOM so that ϕ_m_ = 0.92 (*40*). With these simplifying calibrations, the Kerr rotation signal can be expressed as (see section S2 for a full derivation)

$$\theta_{k}\approx -\frac{1}{2}\;\arctan\left[0.543\frac{V_{\mathrm{APD}}^{1\omega }}{V_{\mathrm{APD}}^{2\omega }}\right]$$ (10)

where $V_{\mathrm{APD}}^{1\omega }$ ($V_{\mathrm{APD}}^{2\omega }$) is the APD voltage measured at the first (second) harmonic of the EOM frequency. We quantify our Kerr rotation noise to be less than $5\;\mu \mathrm{rad}/\sqrt{\mathrm{Hz}}$ using a low power density on the sample (2 μW/μm^2^), comparable to the noise in (*40*) with the similar average power on the APD detector (∼1 μW). The low power ensures that the laser does not substantially heat the sample. More details can be found in sections S2 and S3.

## Supplementary Materials

### This PDF file includes:

Sections S1 to S6 Figs. S1 to S8 References
