# Supplementary material for: Sagnac interferometry for high-sensitivity optical measurements of spin-orbit torque
Source: Sci Adv. 2023 Sep 8;9(36):eadi9039. doi: 10.1126/sciadv.adi9039 (PMC10491211; doi:10.1126/sciadv.adi9039)
Supplement: Supplementary file 1 — Sections S1 to S6 Figs. S1 to S8 References [file sciadv.adi9039_sm.pdf]

Science Advances
AAAS

Supplementary Materials for

Sagnac interferometry for high-sensitivity optical measurements of spin-orbit torque

Saba Karimeddiny *et al.*

Corresponding author: Daniel C. Ralph, dcr14@cornell.edu; Yunqiu Kelly Luo, kelly.y.luo@usc.edu

Sci. Adv. **9**, eadi9039 (2023)
DOI: 10.1126/sciadv.adi9039

This PDF file includes:

Sections SI to SVI
Figs. S1 to S8
References

I. Details of the Sagnac Interferometer

We begin this section by recommending the work by Fried et al., Rev. Sci. Instrum. **85**, 103707 (2014) (*39*). This paper served as the most helpful resource when building and debugging our interferometer, and many of the details in this section are inspired by the helpful level of detail in that work. We will show again here Fig. 1 from our main, but we will discuss some of the finer details of the apparatus. The entire setup, including all of the optics, sample stage,

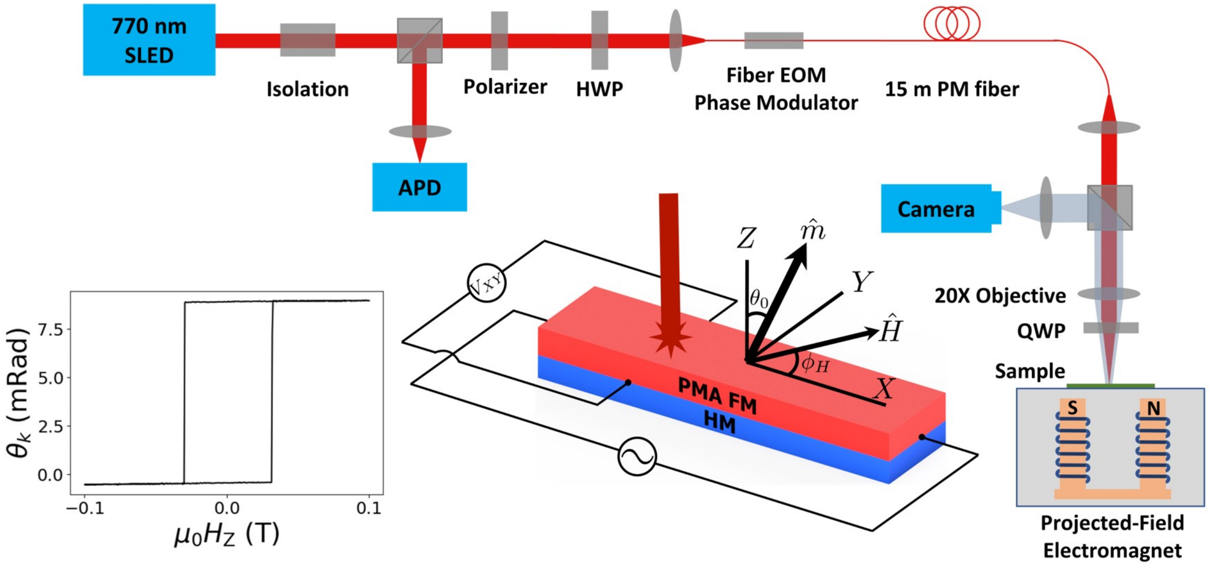

Figure S1: **Schematic of the Sagnac interferometer.** Main text Fig. 1 repeated here for ease of viewing.

and magnet are housed on a floating optical table and enclosed in a rigid polycarbonate box affixed with sound-proof foam to block air currents and external vibrations.

For the source, we use a 770 nm SLED, which has a broad ($\approx$ 15 nm) linewidth. In our original design we used an ultra-narrow-linewidth 780 nm diode, but we found that the broad-linewidth source reduced our noise by about a factor of two; this is because the small-linewidth source has a long beam coherence length. Therefore, the forward-going beam remained coherent with the reflected beam upon cycling through the apparatus, which gave them the opportunity to interfere and produce spurious interference signals not related to the Kerr rotation. The SLED diode and most of its pigtailed fiber are stored inside a closed styrofoam box within

the polycarbonate box to further prevent temperature fluctuations and air currents. We use two Faraday isolators that provide > 65 dB of isolation to protect the diode from backreflections. Backreflections into the diode affect not only the longevity of the diode itself, but also can cause spurious intensity/spectral variations.

Next, the EOSpace fiber electro-optic phase modulator (EOM) is driven by a 50-MHz-bandwidth Zurich Instruments HF2LI lock-in amplifier and all of the signals (transport and optics) are detected on the same lock-in using its multiple demodulators. Our EOM is permanently pigtailed with a 5 m fiber and we append a 10 m fiber to it for a total length of 15 m. Both the EOM and the fiber are stored inside a closed styrofoam box (not the same box as the diode) within the polycarbonate box.

Upon exiting the fiber above the objective stage, the beam is collimated by a screw-on FC/APC lens adapter to a beam diameter of about 8 mm. We choose such a large beam diameter to maximize the filling of the back aperture of the objective lens and reduce our beam spot size on the sample. The beamsplitter after the collimating lens is retractable. It is illuminated with white light and inserted only to align the desired sample properly under the beam. The beam does not go through this beamsplitter during measurement. For the objective we choose to use a $20\times$ near-IR ultra-long-working-distance objective lens to minimize the spot size, maximize the numerical aperture and field-of-view, and leave enough room for probes to make contact to the sample. The quarter-wave plate (QWP) is placed *after* the lens so that light is still linearly polarized while going through the lens. Most lenses have non-negligible Verdet constants so this is very important for reducing the spurious Faraday rotation incurred by the beam while it traverses the lens.

Our beam spot size on the sample is approximately 6 μm and our power incident on the device is < 70 μW. We find that optical powers exceeding a few hundred μW can begin to show local heating effects on the sample as indicated, e.g., as a change in the magnetic coercivity. To accommodate such a low-power beam, we detect the signal with a 50-MHz-bandwidth avalanche photodiode (APD) because it maintains a very low noise equivalent power (NEP) while sacrificing its saturation power, which we remain safely below.

II. Derivation of the Sagnac MOKE Signal

A. Measurement of the Kerr rotation angle θ_k in the absence of applied current

We will use the language of Jones matrices to derive Eq. 1 in the main text. This formalism allows us to calculate the behavior of two orthogonal modes of light at the same time. First, we define some general Jones matrices:

$$\mathrm{P}(\theta_p) = \begin{pmatrix} \cos^2\theta_p & \sin\theta_p\cos\theta_p \\ \sin\theta_p\cos\theta_p & \sin^2\theta_p \end{pmatrix} \tag{11}$$

$$\mathrm{WP}(\theta_{wp},\phi_{wp}) = \begin{pmatrix} \cos\frac{\phi_{wp}}{2} + i\sin\frac{\phi_{wp}}{2}\cos 2\theta_{wp} & i\sin\frac{\phi_{wp}}{2}\sin 2\theta_{wp} \\ i\sin\frac{\phi_{wp}}{2}\sin 2\theta_{wp} & \cos\frac{\phi_{wp}}{2} - i\sin\frac{\phi_{wp}}{2}\cos 2\theta_{wp} \end{pmatrix} \tag{12}$$

$$\mathrm{EOM}(t) = \begin{pmatrix} e^{i\phi_{\parallel}\sin\omega t} & 0 \\ 0 & e^{i\phi_{\perp}\sin\omega t} \end{pmatrix} \tag{13}$$

$$\mathrm{S} = \frac{1}{2}\begin{pmatrix} \frac{e^{-i\delta_+}}{r_+} + \frac{e^{-i\delta_-}}{r_-} & i\left(\frac{e^{-i\delta_+}}{r_+} - \frac{e^{-i\delta_-}}{r_-}\right) \\ -i\left(\frac{e^{-i\delta_+}}{r_+} - \frac{e^{-i\delta_-}}{r_-}\right) & \frac{e^{-i\delta_+}}{r_+} + \frac{e^{-i\delta_-}}{r_-} \end{pmatrix}. \tag{14}$$

Here, our Jones vectors are in the basis of the laboratory: $\mathrm{P}(\theta_p)$ is a polarizer oriented at an angle θ_p. $\mathrm{WP}(\theta_{wp},\phi_{wp})$ is a ϕ_{wp}-wave plate oriented at an angle θ_{wp}. EOM is the electro-optical phase modulator that applies a voltage-dependent phase ($\phi_\perp$ or $\phi_\parallel$ depending on whether the polarization of the incoming beam is along or perpendicular-to the optical axis of the EOM crystal) at a frequency of ω. In the main text we say that the EOM only applies the phase to the beam traveling along the slow axis of the fiber; this is how the EOMs are designed, but our Jones matrix is more general to account for some phase shifts in the fast-axis beam, as well. Our final result is unchanged by this. S is the effect of the sample, which quite generally, has left- and right-circularly polarized light as its eigenvectors and applies an unequal phase ($\delta_+ \neq \delta_-$; “circular birefringence”) and an unequal Fresnel reflectance ($r_+ \neq r_-$; “circular dichroism”) to each of the two helicities of light. The effect of the sample reflectance exchanging the handedness of circularly polarized light is not captured by S, but will rather be accomplished by a complex conjugation later.

At the start of the beam path for the interferometer, unpolarized light exits our laser and encounters a polarizer, P, oriented such the power lost through cross-polarization of the source

beam is minimized (the source diode outputs partially-polarized light). We will assume without loss of generality that polarizer angle is 0° so the starting point for our Jones calculus is

$$v = \begin{pmatrix} 1 \\ 0 \end{pmatrix}.$$

From our beam path we can simply apply the time-ordered Jones matrices of our optical components:

$$\text{P}(0)\text{WP}(\pi/8,\pi)\text{EOM}(t+\tau)\text{WP}(\pi/4,\pi/2)\left[\text{S}\,\text{WP}(\pi/4,\pi/2)\text{EOM}(t)\text{WP}(\pi/8,\pi)v\right]^*. \quad (15)$$

In words, we begin with linearly-polarized light that is polarized at 0° (v). The beam goes through a half-wave plate that rotates the polarization of the beam to 45°, which is equivalent to to two superimposed beams, one horizontally polarized and one vertically polarized. Subsequently, the light goes through an EOM at time t, then through a quarter-wave plate, reflects from the sample, the LCP and RCP beams exchange due to the reflection (this is captured by the complex conjugation), goes through the quarter-wave plate again, through the EOM at a (now later) time $t+\tau$, and finally through the polarizer. We define τ as the time it takes for the beam to travel from the EOM to the sample and back. The result of the above matrix product is

$$\left(\frac{ie^{-i\delta_- + \phi_\| \sin\omega t + \phi_\perp \sin[\omega(t+\tau)]}}{2\,r_-} + \frac{ie^{-i\delta_+ + i\phi_\perp \sin\omega t + \phi_\| \sin[\omega(t+\tau)]}}{2\,r_+}\right)\begin{pmatrix} 1 \\ 0 \end{pmatrix}. \quad (16)$$

In our experiment, we specifically tune the EOM frequency, ω, such that $\tau = \pi/\omega$ (*38, 39*); this results in the simplification:

$$\left(\frac{ie^{-i\delta_- + i\phi_m \sin\omega t}}{2\,r_-} + \frac{ie^{-i\delta_+ - i\phi_m \sin\omega t}}{2\,r_+}\right)\begin{pmatrix} 1 \\ 0 \end{pmatrix} \quad (17)$$

where ϕ_m is the modulation depth $\phi_m := \phi_\| - \phi_\perp$. We detect the time-averaged intensity of light so we take half of the complex square of the above to get:

$$\frac{1}{8\,r_-^2} + \frac{1}{8\,r_+^2} + \frac{1}{8\,r_- r_+}\left(e^{i(\delta_+ - \delta_-)}e^{2i\phi_m \sin\omega t} + e^{-i(\delta_+ - \delta_-)}e^{-2i\phi_m \sin\omega t}\right). \quad (18)$$

Next, we define

$$\theta_k := (\delta_+ - \delta_-)/2$$

Note θ_k is the angle linearly polarized light would rotate after hitting the sample, as one can see by applying the sample matrix (Eq. 14) to any linearly polarized Jones vector.

Applying this definition and the Jacobi-Anger expansion to the light-intensity expression, we get

$$\frac{1}{8\,r_-^2} + \frac{1}{8\,r_+^2} + \frac{1}{8\,r_- r_+}\left(e^{2i\theta_k}\sum_{n=-\infty}^{\infty} J_n(2\phi_m)e^{in\omega t} + e^{-2i\theta_k}\sum_{n=-\infty}^{\infty} J_n(2\phi_m)e^{-in\omega t}\right). \tag{19}$$

To measure the first harmonic signal, we use a lock-in amplifier to isolate the component proportional to $\sin(\omega t)$

$$\begin{aligned}
I^\omega =& \frac{1}{T}\int_T dt\left[\frac{1}{8\,r_-^2} + \frac{1}{8\,r_+^2} + \frac{1}{8\,r_- r_+}\left(e^{2i\theta_k}\sum_{n=-\infty}^{\infty} J_n(2\phi_m)e^{in\omega t} + e^{-2i\theta_k}\sum_{n=-\infty}^{\infty} J_n(2\phi_m)e^{-in\omega t}\right)\right]\sin\omega t \\
=& \frac{1}{2iT}\int_T dt\left[\frac{1}{8\,r_- r_+}\left(e^{2i\theta_k}\sum_{n=-\infty}^{\infty} J_n(2\phi_m)e^{in\omega t} + e^{-2i\theta_k}\sum_{n=-\infty}^{\infty} J_n(2\phi_m)e^{-in\omega t}\right)\right]\left[e^{i\omega t} - e^{-i\omega t}\right] \\
=& \frac{1}{2iT}\int_T dt\left[\frac{1}{8\,r_- r_+}\left(e^{2i\theta_k}\sum_{n=-\infty}^{\infty} J_n(2\phi_m)\left(e^{i(n+1)\omega t} - e^{i(n-1)\omega t}\right)\right.\right. \\
& \left.\left. + e^{-2i\theta_k}\sum_{n=-\infty}^{\infty} J_n(2\phi_m)\left(e^{-i(n-1)\omega t} - e^{-i(n+1)\omega t}\right)\right)\right].
\end{aligned} \tag{20}$$

The only terms in the sums that will survive the integration are those for which the complex time-dependent exponentials are identically 1 (i.e. when $n+1=0$ or $n-1=0$):

$$I^\omega = \frac{1}{2iT}\int_T dt\left[\frac{1}{8\,r_- r_+}\left[e^{2i\theta_k}\left(J_{-1}(2\phi_m) - J_1(2\phi_m)\right) + e^{-2i\theta_k}\left(J_1(2\phi_m) - J_{-1}(2\phi_m)\right)\right]\right] \tag{21}$$

$$= \frac{1}{T}\int_T dt\frac{1}{8\,r_- r_+}\left[\sin 2\theta_k\left(J_{-1}(2\phi_m) - J_1(2\phi_m)\right)\right] \tag{22}$$

$$= \frac{1}{8\,r_- r_+}\left[\sin 2\theta_k\left(J_{-1}(2\phi_m) - J_1(2\phi_m)\right)\right] \tag{23}$$

$$= -\frac{\sin 2\theta_k J_1(2\phi_m)}{4\,r_- r_+}. \tag{24}$$

Here we applied the $J_{-1} = -J_1$ property of the Bessel-J functions. We can compute the second harmonic (the $\cos 2\omega t$ component) using an analogous procedure

$$I^{2\omega} = \frac{\cos 2\theta_k J_2(2\phi_m)}{4\, r_- r_+}. \tag{25}$$

From these two expressions, we can solve for θ_k and also normalize out all of the dependencies on the Fresnel amplitude coefficients (r_+ and r_-) by simply taking the ratio of the two signals:

$$\theta_k = -\frac{1}{2} \arctan\left[\frac{J_2(2\phi_m) I^{\omega}}{J_1(2\phi_m) I^{2\omega}}\right]. \tag{26}$$

For our measurements, we maximize the first harmonic signal, because it is proportional to the quantity we want to measure (θ_k). By tuning ϕ_m to maximize $J_1(2\phi_m)$, we get $\phi_m = 0.92$ (*39*) and $J_2(2\phi_m)/J_1(2\phi_m) \approx 0.543$. The above equation and aforementioned constant are exactly Eq. 1 in the main text.

B. Measurement of changes in the Kerr angle $\Delta\theta_k$ due to current-induced magnetic deflections

To derive a similar result with an AC applied current, we can begin at Eq. (19) with an added oscillation from a time-dependent θ_k that results from current-induced tilting of the magnetic moment at the current frequency ω_e:

$$\frac{1}{8\, r_- r_+}\left(e^{2i(\theta_k + \Delta\theta_k \sin\omega_e t)} \sum_{n=-\infty}^{\infty} J_n(2\phi_m) e^{in\omega t} + e^{-2i(\theta_k + \Delta\theta_k \sin\omega_e t)} \sum_{n=-\infty}^{\infty} J_n(2\phi_m) e^{-in\omega t}\right). \tag{27}$$

We can apply the Jacobi-Anger expansion again

$$\frac{1}{8\, r_- r_+}\left(e^{2i\theta_k} \sum_{n,m=-\infty}^{\infty} J_n(2\phi_m) J_m(2\Delta\theta_k) e^{i(n\omega + m\omega_e)t} + e^{-2i\theta_k} \sum_{n,m=-\infty}^{\infty} J_n(2\phi_m) J_m(2\Delta\theta_k) e^{-i(n\omega + m\omega_e)t}\right). \tag{28}$$

Now we demodulate this signal at the sideband frequency $\omega \pm \omega_e$. We will only show the $\omega + \omega_e$ derivation for sign simplicity, but the result is identical for the upper and lower sidebands:

$$
\begin{aligned}
I^{\omega+\omega_e} =& \frac{1}{T}\int_T dt \frac{1}{8\, r_- r_+}\left(e^{2i\theta_k} \sum_{n,m=-\infty}^{\infty} J_n(2\phi_m) J_m(2\Delta\theta_k) e^{i(n\omega + m\omega_e)t} + \right. \\
& \left. e^{-2i\theta_k} \sum_{n,m=-\infty}^{\infty} J_n(2\phi_m) J_m(2\Delta\theta_k) e^{-i(n\omega + m\omega_e)t} \right) \times \cos(\omega t + \omega_e t) \\
=& \frac{1}{2T}\int_T dt \frac{1}{8\, r_- r_+}\left(e^{2i\theta_k} \sum_{n,m=-\infty}^{\infty} J_n(2\phi_m) J_m(2\Delta\theta_k) e^{i(n\omega + m\omega_e)t} + \right. \\
& \left. e^{-2i\theta_k} \sum_{n,m=-\infty}^{\infty} J_n(2\phi_m) J_m(2\Delta\theta_k) e^{-i(n\omega + m\omega_e)t} \right) \times \left(e^{\omega t + \omega_e t} + e^{-\omega t - \omega_e t} \right)
\end{aligned}
\tag{29}
$$

Again, the only complex exponentials that will survive integration are the ones where the exponent is identically zero. This leaves us with:

$$
\begin{aligned}
I^{\omega+\omega_e} =& \frac{1}{16\, r_- r_+}\left[e^{2i\theta_k}\left(J_{-1}(2\phi_m) J_{-1}(2\Delta\theta_k) + J_1(2\phi_m) J_1(2\Delta\theta_k) \right) + \right. \\
& \left. e^{-2i\theta_k}\left(J_{-1}(2\phi_m) J_{-1}(2\Delta\theta_k) + J_1(2\phi_m) J_1(2\Delta\theta_k) \right) \right] \\
=& \frac{1}{4\, r_- r_+} \cos 2\theta_k J_1(2\phi_m) J_1(2\Delta\theta_k).
\end{aligned}
\tag{30}
$$

In our experiments $\Delta\theta_k$ is very small so we use that $J_1(x) \approx x/2$ for small x

$$
I^{\omega+\omega_e} = \frac{\cos 2\theta_k J_1(2\phi_m)}{4\, r_- r_+} \Delta\theta_k. \tag{31}
$$

Finally, we take the ratio of this signal with the second harmonic (at ω) derived earlier to reach a simple expression for the current-induced change in the Kerr signal

$$
\Delta\theta_k = \frac{J_2(2\phi_m) I^{\omega+\omega_e}}{J_1(2\phi_m) I^{2\omega}}. \tag{32}
$$

All of the $\Delta\theta_k$ data presented are determined using this equation.

III. Absence of Quadratic MOKE effects

Quadratic MOKE (qMOKE) is a magneto-optic effect that is second-order in magnetization, specifically, the in plane moments. This section justifies analytically and experimentally

our main-text claim that qMOKE negligibly impacts our Sagnac signals, despite appearing in conventional polar-Kerr rotation measurements. In those measurements, linearly polarized light illuminates the sample at normal incidence. Upon reflection, the polarization rotates by (*28*)

$$\theta_{k,linear} = \kappa m_z + \beta_Q m_x m_y \tag{33}$$

where κ is still the MOKE coupling parameter, β_Q is the qMOKE coupling parameter and $\hat{m}$ is the magnetization unit vector. The components of $\hat{m}$ are defined in coordinates such that z is still the film normal, but now x lies along the plane of light polarization.

In contrast, the next section derives the Sagnac signal to be

$$\theta_{k,Sag} = \kappa m_z + 2\beta_Q m_x m_y \sin(\kappa m_z)\left[0.71\cos(2\kappa m_z) - 0.62\cos^2(\kappa m_z)\right] + O\left(\beta_Q^2\right). \tag{34}$$

The first term is equivalent to $\theta_k := (\delta_+ - \delta_-)/2$ defined previously. The second term comes from qMOKE. However, unlike $\theta_{k,linear}$, the ratio of the qMOKE term to the polar MOKE term is only of order $\beta_Q\kappa/\kappa = \beta_Q$ (not β_Q/κ). Since β_Q is of order 10^{-4} (*28*), the qMOKE contribution should be negligible compared to the polar MOKE contribution to the Sagnac Signal.

A. Calculation of the qMOKE contribution

The effect of qMOKE on the Sagnac signal can be derived by extending our Jones matrix calculation (Eq. 15), with two changes: a new sample matrix and mirror operator:

$$\mathrm{P}(0)\mathrm{WP}(-\pi/8,\pi)\mathrm{EOM}(t+\tau)\mathrm{WP}(-\pi/4,\pi/2)\mathrm{mirror}\left[\mathrm{S}_Q\,\mathrm{WP}(\pi/4,\pi/2)\mathrm{EOM}(t)\mathrm{WP}(\pi/8,\pi)v\right]. \tag{35}$$

First, we replace the sample reflection matrix S used previously, with a new sample reflection matrix S_Q that includes the quadratic effects. We start with our original sample matrix (Eq. 14), and for simplicity assume no circular dichroism ($\xi := 1/r_+ = 1/r_-$) . We set the phase shifts to be equal ($\delta_+ = -\delta_- = \kappa m_z$) without loss of generality, because unequal phase shifts only cause a global phase, which is irrelevant and also can be absorbed into the prefactor ξ. Finally we add in the quadratic effect term, following Fan et al. (*28*). For the coordinate

frame where the in-plane magnetization points along the x direction, the sample matrix including quadratic MOKE can be written

$$M_k = \xi \begin{pmatrix} \cos(\kappa m_z) + \frac{1}{2}\beta_Q \sin^2(\theta) & \sin(\kappa m_z) \\ -\sin(\kappa m_z) & \cos(\kappa m_z) - \frac{1}{2}\beta_Q \sin^2(\theta) \end{pmatrix} \tag{36}$$

where θ is the polar angle of the magnetization. Note that this matrix is identical to Fan et al. (*28*) to lowest order in κ and β_Q. Next, because the magnetic moment may point in other directions besides the x-z-plane we change the basis of M_K by a rotation about the z axis (*28*). To do this, we apply the rotation matrix R, defined as

$$R(\phi) = \begin{bmatrix} \cos\phi & -\sin\phi \\ \sin\phi & \cos\phi \end{bmatrix}, \tag{37}$$

where ϕ is the azimuthal angle of the magnetization, to yield the general sample reflection matrix (in the linearly-polarized laboratory basis)

$$\begin{aligned} S_Q &= R(\phi) \cdot M_K \cdot R(-\phi) \\ &= \xi \times \begin{pmatrix} \cos(\kappa m_z) + \frac{1}{2}\beta_Q \sin^2(\theta)\cos(2\phi) & \sin(\kappa m_z) + \beta_Q \sin^2(\theta)\sin(\phi)\cos(\phi) \\ \beta_Q \sin^2(\theta)\sin(\phi)\cos(\phi) - \sin(\kappa m_z) & \cos(\kappa m_z) - \frac{1}{2}\beta_Q \sin^2(\theta)\cos(2\phi) \end{pmatrix} \end{aligned} \tag{38}$$

Note that for ferromagnets with PMA or easy-plane anistropy where the in-plane magnetization follows the in-plane applied field such as in our case: $\phi = \phi_{\mathrm{H}} + \phi_{\mathrm{frame}}$. ϕ_{frame} is added to account for the arbitrary rotation of the reference frame of light polarization relative to the current direction due to the fiber. The above equation can also be expressed equivalently in terms of the Cartesian components of the magnetization unit vector:

$$S_Q = \xi \times \begin{pmatrix} \cos(\kappa m_z) + \frac{1}{2}\beta_Q \left(m_x^2 - m_y^2\right) & m_x m_y \beta_Q + \sin(\kappa m_z) \\ m_x m_y \beta_Q - \sin(\kappa m_z) & \cos(\kappa m_z) - \frac{1}{2}\beta_Q \left(m_x^2 - m_y^2\right) \end{pmatrix}. \tag{39}$$

The second change between Eq. (15) and Eq. (35) is that the mirror operator replaces the complex conjugation. qMOKE requires this generalization because the complex conjugate only mirrors circularly polarized light, not linearly polarized light. qMOKE produces linear light components, even when illuminated with circular light, so it is necessary to mirror those components upon reflection as well. The mirror operator is

$$\text{mirror} = \begin{pmatrix} 1 & 0 \\ 0 & -1 \end{pmatrix}. \tag{40}$$

which flips the sign of the y-component of the electric field. This choice of mirroring axis is physically irrelevant; it only causes a global phase shift and changes the angles of the optical elements through which the light back-propagates. We chose to mirror over the x axis, so that the angles of the optical elements simply pick up a minus sign.

The above two changes to the Jones matrices result in the following Jones vector at the detector:

$$-i\xi\left(\cos\left(\phi_m \sin(tw)+\kappa m_z\right)+\frac{1}{2}\beta_Q \sin^2(\theta)\sin(2\phi)\right)\begin{pmatrix}1\\0\end{pmatrix}. \tag{41}$$

The first term here matches the previously calculated version (Eq. (17)), within the assumptions we made. The second term represents the quadratic effects, which have no dependence on the time-dependent phase modulation from the electro-optic modulator. If we were able to make a direct measurement of the modulated part of the electric field, qMOKE would give no contribution. However, we measure the intensity, so the qMOKE signal contributes to the modulated intensity upon multiplying with the other term while taking the complex square. Nevertheless, this results in a much smaller contribution to the Sagnac signal from qMOKE than might be anticipated intuitively.

Now that we have the Jones vector incident on the detector, we repeat the mathematics of section A. to derive the new Sagnac signal:

$$\theta_{k,Sag}=\frac{1}{4}\tan^{-1}\left(\frac{2\phi_m J_2(2\phi_m)\sin(\kappa m_z)\left(\beta_Q J_1(\phi_m)\sin^2(\theta)\sin(2\phi)+J_1(2\phi_m)\cos(\kappa m_z)\right)}{|\phi_m|\,J_1(2\phi_m)\left(2\beta_Q J_2(\phi_m)\sin^2(\theta)\sin(2\phi)\cos(\kappa m_z)+J_2(2\phi_m)\cos(2\kappa m_z)\right)}\right). \tag{42}$$

In the case of no quadratic effects ($\beta_Q = 0$), this expression reduces to the first order Kerr rotation:

$$\theta_{k,Sag}=m_z\kappa. \tag{43}$$

We can also expand the full expression for $\theta_{k,Sag}$ to first order in β_Q:

$$\theta_{k,Sag}=\kappa m_z+\beta_Q\sin^2(\theta)\sin(2\phi)\sin(\kappa m_z)\left(\frac{J_1(\phi_m)\cos(2\kappa m_z)}{J_1(2\phi_m)}-\frac{2J_2(\phi_m)\cos^2(\kappa m_z)}{J_2(2\phi_m)}\right)+O\left(\beta_Q^2\right) \tag{44}$$

and, upon substituting in the modulation depth $\phi_m = 0.92$ used for the Sagnac measurement,

$$\theta_{k,Sag}=\kappa m_z+\beta_Q\sin^2(\theta)\sin(2\phi)\sin(\kappa m_z)\left(0.71\cos(2\kappa m_z)-0.62\cos^2(\kappa m_z)\right)+O\left(\beta_Q^2\right). \tag{45}$$

From this we conclude that a qMOKE signal would have a dependence on the angle of the in-plane magnetization $\propto m_x m_y \propto \sin(2\phi) \propto \sin(2\phi_{\rm H})$, and we have reached the expression of Eq. (34).

B. Experimental limit on the qMOKE contribution

To test experimentally for any contribution of qMOKE to the Sagnac signal, we perform a measurement analogous to a calibration of the electrical planar Hall effect – we directly measure the change in the Sagnac signal as we apply an in-plane magnetic field to tilt the PMA magnet partially in-plane and then rotate the field angle ϕ_H. Supplementary Fig. S2 shows the result of these measurements for (a) the electrical Hall signal and (b) the Sagnac signal, each for 3 different strengths of applied magnetic field. The data for this figure were collected on the same Pt(4 nm)/Co(1.15 nm) sample discussed in the main text (i.e, Figs. 1-2), which had $\mu_0 M_{\rm eff} \approx -0.42$ T, as calculated by the parabolic fits in the main text.

As explained above (Eq. (34)), if there were any measurable contribution from qMOKE, we should expect a signal $\propto \sin(2\phi_{\rm H})$, i.e., with a π periodicity. Such a π-periodic signal is clearly visible in the electrical planar Hall measurement. However the $\phi_{\rm H}$ dependence of the Sagnal signal is much weaker, and it is 2π-periodic, not π-periodic. Therefore, no contribution from qMOKE is measurable.

Based on the calculation above (Eq. (34) or (45)), with the parameters $\kappa = 4.9 \times 10^{-3}$ (from the hysteresis curve in Fig. 1 of the main text), $\beta_Q = 1.1 \times 10^{-4}$ (based on data for Pt/Py from X. Fan et al. (*28*)), and $\theta = \arcsin(H/|M_{\rm eff}|)$, the expected amplitude of the $\sin(2\phi_{\rm H})$ signal for the $|\mu_0 H|$ = 75 mT scan is approximately 1.4 nano-radians. This is indeed orders of magnitude less than the experimental noise in Fig. S2(b), so in agreement with the experiment we should not expect any visible qMOKE contribution.

The π-periodic signal that is visible in Fig. S2(b) can be understood instead as due to a small misalignment of the applied magnetic field from the plane of the sample. The first-harmonic

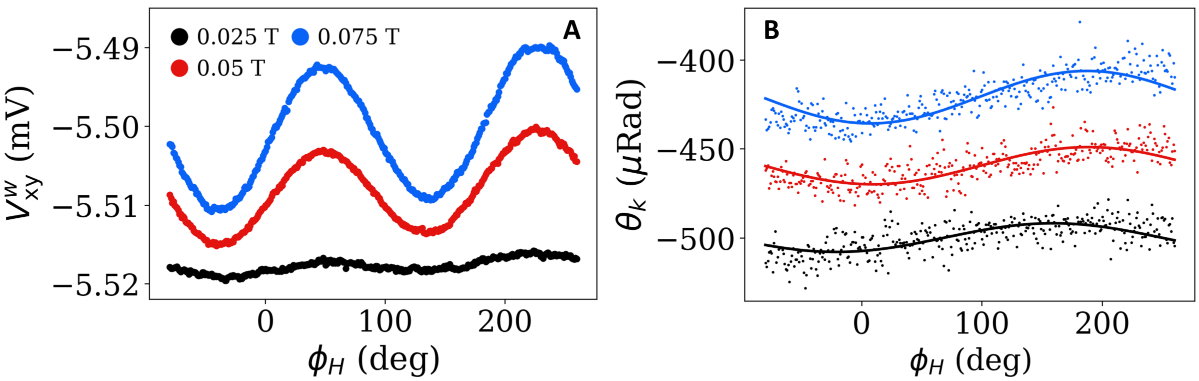

Figure S2: **The electrical Hall signal and the Sagnac signal for sweeps of in-plane field angle ϕ_H.** (**A**) The measured first-harmonic electrical Hall signal, V^{ω}_{XY} due to a combination of the anomalous and planar Hall effects and (**B**) the Kerr rotation signal measured by the Sagnac interferometer, θ_k, vs. the angle of applied magnetic field within the plane of the magnet. Three different strengths of applied magnetic field are shown and they are artificially vertically offset in (**B**) for clarity. The overlayed lines are best-fits to Eq. (47). The data are collected for the Pt(4 nm)/Co(1.15 nm) sample with $\mu_0 M_{\text{eff}} \approx -0.42$ T; this is the same device for which measurements are highlighted in the main text Fig. 2.

Hall voltage signal as a function for small tilt angles has the form (*6, 35*)

$$\begin{aligned}\frac{V^{\omega}_{XY}}{\Delta I} =& R_{\text{AHE}} \cos\left(\frac{H}{|M_{\text{eff}}|}\right) \\ &+ R_{\text{PHE}} \sin^2\left(\frac{H}{|M_{\text{eff}}|}\right) \sin\phi_H \cos\phi_H \\ &+ R_{\text{AHE}} \frac{H^2 \sin\theta_{\text{off}}}{(M_{\text{eff}})^2} \sin\left(\frac{H}{|M_{\text{eff}}|}\right) \cos(\phi_H - \phi_{\text{off}}). \end{aligned} \tag{46}$$

In analogy with Hall measurements, for a field rotation axis misaligned by an angle θ_{off} relative to the sample normal direction, the polar-MOKE Sagnac signal should have the dependence for small tilts (*35*)

$$\theta_k \approx \kappa \cos\left(\frac{H}{|M_{\text{eff}}|}\right) + \kappa \frac{H^2 \sin\theta_{\text{off}}}{(M_{\text{eff}})^2} \sin\left(\frac{H}{|M_{\text{eff}}|}\right) \cos(\phi_H - \phi_{\text{off}}). \tag{47}$$

Fits of these curves for both the Hall and Sagnac measurements are shown in Supplementary Fig. S2. Both sets of data indicate a field/sample tilt of $\theta_{\text{off}} \sim 1^{\circ}$ (Supplementary Fig. S3). This is most likely caused by a slight misalignment of the projected-field magnet (GMW 5201) center.

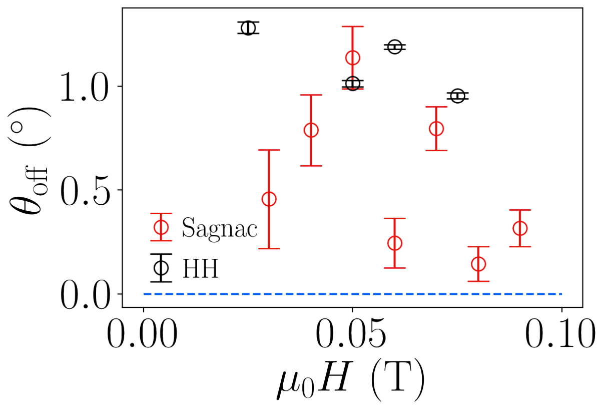

Figure S3: **Field misalignment angle calibration.** The measured field misalignment angle, $\theta_{\rm off}$, versus the strength of applied magnetic field applied nominally in the plane of the device. $\theta_{\rm off} = 0°$ means that the magnetic field is perfectly aligned in the device plane.

IV. Expression of Kerr rotation θ_K and current induced changes in the Kerr angle $\Delta\theta_K$.

In this section, we will derive the field dependence of the Kerr rotation and current induced changes in the Kerr angle, coupled to just the out-of-plane component of the magnetic moment. To do this, we follow a procedure similar to those used in refs. (*6, 35*) for deducing the equilibrium positions and current-induced modulation amplitudes of the magnetization.

We begin the derivation by writing the equilibrium magnetic energy divided by the total magnetic moment in the absence of any applied current

$$\begin{aligned}\frac{F_{\rm eq}(\theta,\phi)}{M_s} &= -\mu_0 \mathbf{m}\cdot\mathbf{H} + \frac{\mu_0 M_{\rm eff}}{2}(\mathbf{m}\cdot\hat{Z})^2 \\ &= -\mu_0 H \sin\theta \sin\theta_H \cos(\phi-\phi_H) - \frac{\mu_0}{2}\cos\theta(2H\cos\theta_H - M_{\rm eff}\cos\theta). \qquad (48)\end{aligned}$$

Here $F_{\rm eq}$ is the equilibrium free energy, M_s is the saturation magnetization, $\mathbf{m}$ is the normalized vector magnetic moment, $\mathbf{H}$ is the vector external magnetic field, and $\mu_0 M_{\rm eff} = \mu_0 M_s - 2K_\perp/M_s$ is the effective magnetization. PMA is indicated by a negative $M_{\rm eff}$. The angles in the second line denote the direction of external applied magnetic field when subscripted with an H and refer to the the direction of the magnetic moment when they lack a subscript. Minimization of this free energy yields the equilibrium magnetic orientation θ_0,ϕ_0. As we apply an AC current the SOTs produced will act as effective fields that reorient the magnetic moment. This is a

"slow" process ($\dot{\mathbf{m}} \ll \gamma|H|$) so it may be described as an effective modification of equilibrium free energy (Eq. (48)). With the perturbation from a general, current-induced effective magnetic field, $\Delta\mathbf{H}$ (assumed small compared to H), the free energy becomes

$$\begin{aligned}\frac{F(\theta,\phi)}{M_s} =& \frac{F_{\text{eq}}(\theta,\phi)}{M_s} - \mu_0 \mathbf{m}\cdot\Delta\mathbf{H} \\ \approx& \frac{F_{\text{eq}}(\theta_0,\phi_0)}{M_s} + \frac{1}{2M_s}\frac{\partial^2 F_{\text{eq}}}{\partial\theta^2}\Big|_{\theta_0,\phi_0}(\Delta\theta)^2 + \frac{1}{2M_s}\frac{\partial^2 F_{\text{eq}}}{\partial\phi^2}\Big|_{\theta_0,\phi_0}(\Delta\phi)^2 + \frac{1}{M_s}\frac{\partial^2 F_{\text{eq}}}{\partial\theta\partial\phi}\Big|_{\theta_0,\phi_0}\Delta\theta\Delta\phi \\ &- \mu_0(\sin\theta\cos\phi\Delta H_X + \sin\theta\sin\phi\Delta H_Y + \cos\theta\Delta H_Z).\end{aligned} \tag{49}$$

(The first derivatives of F_{eq} are zero when evaluated at the equilibrium orientation.) We have included the cross second derivative in this expression, but when evaluated it gives zero.

The new magnetic orientation in the presence of the current-induced magnetic field can then be calculated as a minimization problem

$$\frac{\partial F}{\partial\theta} = \frac{\partial F}{\partial\phi} = 0. \tag{50}$$

Here we consider the case of a PMA magnet when the external field is in-plane ($\theta_H = \frac{\pi}{2}$) and assume negligible within-plane anisotropy so that the in-plane projection of the equilibrium magnetic moment is aligned with the external field i.e. $\phi_0 = \phi_H$.

The solutions of Eq. (50) to first order in the current-induced field yield

$$\Delta\theta = \frac{\cos\theta_0(\Delta H_X\cos\phi_H + \Delta H_Y\sin\phi_H) - \Delta H_Z\sin\theta_0}{-M_{\text{eff}}\cos 2\theta_0 + H\sin\theta_0} \tag{51}$$

$$\Delta\phi = \frac{-\Delta H_X\sin\phi_H + \Delta H_Y\cos\phi_H}{H}. \tag{52}$$

The solution for the equilibrium polar angle of the magnetization is

$$\frac{\partial F_{eq}}{\partial\theta}\Big|_{\theta_0,\phi_0} = -\mu_0 H\cos\theta_0 - \mu_0 M_{\text{eff}}\sin\theta_0\cos\theta_0 = 0 \tag{53}$$

$$\theta_0 = \begin{cases}\arcsin(\frac{H}{|M_{\text{eff}}|}) & H < |M_{\text{eff}}| \\ \frac{\pi}{2} & H \geq |M_{\text{eff}}|.\end{cases} \tag{54}$$

To get from the above equations to the full expected Sagnac MOKE signal (Eqs. (4), (5) and (9) in the main text) we begin with the expression for the polar MOKE signal in the linear regime, which is only sensitive to the out-of-plane component of the magnetization m_z

$$\theta_k = \kappa m_Z. \tag{55}$$

We let θ consist of an equilibrium contribution due to the external magnetic field and a time-dependent contribution due to the AC-current-induced spin-orbit fields: $\theta \to \theta_0 + \Delta\theta$ with $\Delta\theta \ll 1$ and then Taylor expand

$$\theta_k + \Delta\theta_k \approx \kappa\left(\cos\theta_0 - \Delta\theta\sin\theta_0\right). \tag{56}$$

For the tilting measurements on samples with perpendicular magnetic anisotropy, $\sin\theta_0 = H/|M_{\text{eff}}|$ (Eq. 54) and $\phi_0 = \phi_H$. Using the expression for $\Delta\theta$ derived previously (Eq. 51), and separating the equilibrium and current-induced signals in Eq. (56), in the regime of weak applied fields $H \ll |M_{\text{eff}}|$ we get for small tilts about the $\pm$ m_z directions

$$\theta_k = \pm\,\kappa\left(1 - \frac{H^2}{2M_{\text{eff}}^2}\right) \tag{57}$$

$$\Delta\theta_k = \mp\,\kappa\left(\Delta H_X\cos\phi_H + \Delta H_Y\sin\phi_H\right)\frac{H}{M_{\text{eff}}^2}. \tag{58}$$

For in-plane field azimuth sweep measurements on samples with in-plane magnetic anisotropy, $\theta_0 = \frac{\pi}{2}$ and $\phi_0 = \phi_H$. Substituting in the expression for $\Delta\theta$ from Eq. (51), and considering the current-induced term in Eq. (56), we get

$$\begin{aligned}\Delta\theta_K &= \frac{\kappa\Delta H_Z}{H + M_{\text{eff}}}\\ &= \frac{\kappa\Delta H_{DL}\cos\phi}{H + M_{\text{eff}}}.\end{aligned} \tag{59}$$

V. Comparison of a Sagnac MOKE measurement and a conventional polar-MOKE measurement for a PMA thin film

Here we present a comparison between the conventional polar MOKE method and Sagnac MOKE interferometry for Ta/$Co_{40}Fe_{40}B_{20}$ test samples with perpendicular magnetic anisotropy. In Supplementary Fig. S4(a), out-of-plane magnetic hysteresis is measured using a conventional polar MOKE setup for a Ta(4 nm)/$Co_{40}Fe_{40}B_{20}$(0.65 nm) sample. In comparison, Supplementary Fig. S4(b) shows Sagnac MOKE interferometry readout on a similar CoFeB sample from the same wedge wafer with a slightly different thickness Ta(4 nm)/$Co_{40}Fe_{40}B_{20}$(0.85 nm). The difference in the coercivity field between Supplementary Fig. S4(a) and S4(b) is due to this

small difference in film thickness. One can visually observe that the signal-to-noise of the Sagnac MOKE interferometry is a significant improvement compared to conventional polar MOKE. The linear background in polar MOKE readout (Supplementary Fig. S4(a)) comes from the Faraday effect in the objective lens. Sagnac interferometry is insensitive to this effect in the case that the quarter-waveplate is positioned between sample and the objective (*39*). Furthermore in this data, the Sagnac took a single scan in $\sim$ 1 minute with a lock-in amplifier time constant of 10 milliseconds, while the polar-MOKE took the average over 10 scans in a total of $\sim$ 10 - 20 minutes, with a lock-in amplifier time constant of 500 milliseconds.

Our conventional polar-MOKE setup mimics the setup of (*30*) with the only difference of using a Helium-neon laser as the light source. A linearly-polarized beam is incident normal to the sample through an objective lens with a numerical aperture of 0.4, focusing the beam to a circular spot with a full width at half maximum of $\sim$ 1 μm. The rotation of the reflected beam polarization is detected by a balanced photodiode bridge with a noise equivalent power of 1.1 pW/$\sqrt{\text{Hz}}$, which gives a readout noise of $\sim$ 400 μRad/$\sqrt{\text{Hz}}$ for the conventional MOKE setup (Supplementary Fig. S4(a)). In contrast, our Sagnac MOKE readout noise (Supplementary Fig. S4(b)) is less than 5 μRad/$\sqrt{\text{Hz}}$. As we noted in the main text, while conventional MOKE can achieve comparable resolution with external modulation of magnetic field, electric field, or current (*30, 31*), these methods are not applicable for measuring hysteresis curves of ferromagnets.

VI. Pt Sample Details

A. Wedge Thickness

All samples measured are grown by DC-magnetron sputtering onto a high-resistivity, surface-passivated Si/SiO_2 wafer. The stacks are Si/SiO_2/Ta(1.5)/Pt(4)/Co(t_{Co})/MgO(1.9)/Ta(2) where all of the numbers in parentheses are layer thicknesses in nanometers. The bottom Ta is used as a seed layer to promote smooth growth of the films, and the top MgO/Ta stack is used to cap the Co and minimize oxidation of the Co Layer. Both the bottom and capping Ta layers are sufficiently resistive that they carry negligible current density compared to the Pt and Co layers. By strategically stopping the wafer rotation during sputter deposition, we grow the Co

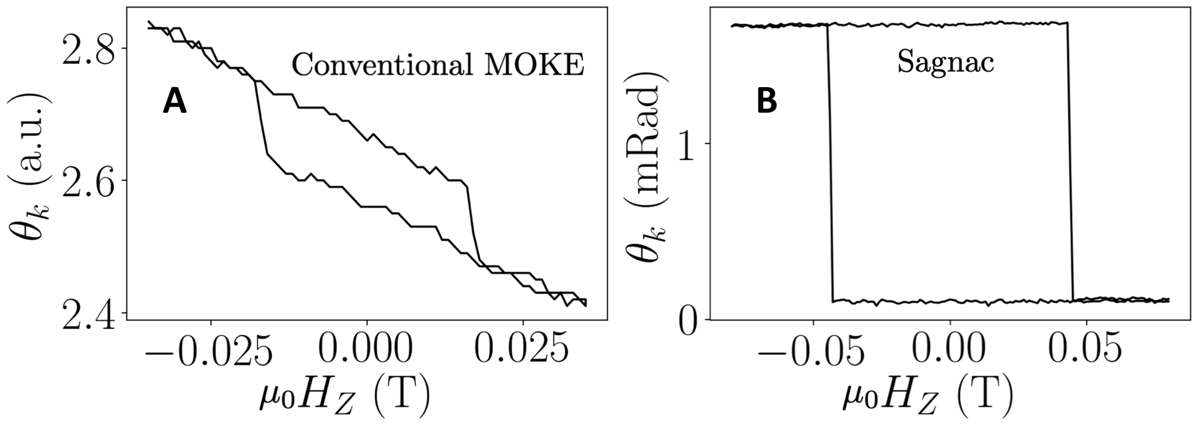

Figure S4: **Comparison between conventional MOKE and Sagnac readouts.** (**A**) Conventional polar MOKE readout compared to (**B**) Sagnac MOKE interferometry readout on a CoFeB film with perpendicular magnetic anisotropy.

layer with a thickness-gradient "wedge". The wedge's thickness gradient is along the direction of current flow (X-axis) for all devices measured. The Co thickness as a function of device distance from the wafer flat is shown in Supplementary Fig. S5, for both PMA series and IP series shown in the main text. This calibration is performed using atomic-force microscopy measurements at different points on a test wafer, followed by a polynomial fit and interpolation to get the thickness variation across the full wafer.

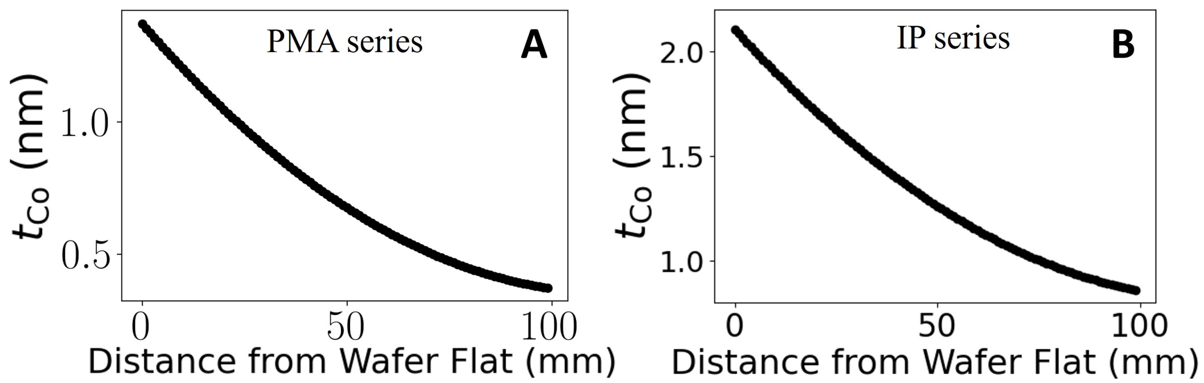

Figure S5: **Co thin wedge film calibration.** The thickness of the Co "wedge" film as a function of the distance from the 4-inch wafer flat for **(A)** PMA series and **(B)** IP series.

B. Film Conductivity

We characterize the electrical conductances of our films by measuring the four-point resistance on many devices across the Co-wedge wafer as shown in Supplementary Fig. S6. In the

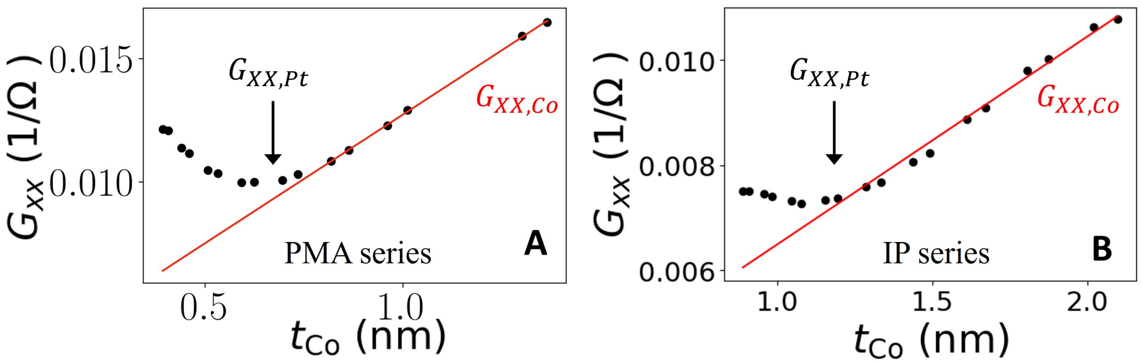

Figure S6: **Electrical conductance measurements.** Measured 4-point conductance of the fabricated devices as a function of the Co film thickness for **(A)** the PMA series and **(B)** the IP series. The red line is a fit to the linear regime of the data where a parallel resistor model of the HM/FM stack is appropriate.

very low Co thickness regime ($\sim$ 0.4 nm), the Co likely does not yet form a continuous film on top of the Pt so we expect the conductance measured here is entirely due to that of a bare Pt. As the Co thickness is increased, the conductance decreases due to increased surface scattering of conduction electrons in the Pt from the growing Co layer. In the regime above 0.8 nm of Co, the conductance is linear in the Co thickness, which is the expected behavior of a simple parallel-resistor model. We fit a line to the linear regime, the slope of which is the (inverse) resistivity of Co: 9.59 μohms cm for the PMA series and 25.26 μohms cm for the IP series. We estimate the resistivity of the 4 nm Pt layer adjacent to an established Co layer as corresponding approximately to the conductivity value at the minimum of the G_{XX} (as indicated in the figure): 40 μohms cm for PMA series and 53.9 μohms cm for IP series. All of the Pt/Co/MgO samples for which we performed measurements of current-induced torque have Co layers thicker than 0.8 nm.

C. Magnetometry

To find the saturation magnetization M_s of the Co, we measure the magnetic moment on 3 mm $\times$ 3 mm thin films diced from the wafer adjacent to the patterned devices with vibrating sample magnetometry (VSM). The magnetic moment it measures is ($\mu_0 M_s$Volume). If we divide this by area, we get $\mu_0 M_s t_{\mathrm{Co}}$, which is an expression we use in the main text Eq. 8. To get this quantity for each device, we plot it versus t_{Co}. t_{Co} and linearly interpolate with the line shown in Supplementary Fig. S7.

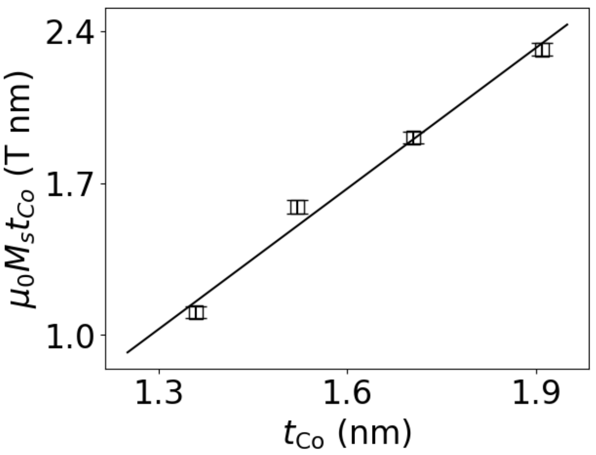

Figure S7: **Saturation magnetization measurements.** Measured saturation magnetization per unit area $\mu_0 M_s t_{\mathrm{Co}}$ as a function of Co thickness t_{Co}.

D. Calibration of effective magnetization of samples with in plane anisotropy via spin-torque ferromagnetic resonance (ST-FMR) measurements

We adopt the conventional ST-FMR measurements to obtain the effective magnetization M_{eff} for devices with in-plane magnetic anisotropy. M_{eff} is obtained by fitting the resonance peak using the Kittel formula (ref. (*41*)). We measured $\mu_0 M_{\text{eff}}$ for eight devices with varying t_{Co} values, as shown below marked in black dots. We then fit the $\mu_0 M_{\text{eff}}$ versus t_{Co} using a second order polynomial, and use the fitted curve to interpolate $\mu_0 M_{\text{eff}}$ for every device with in-plane anisotropy measured in main text Fig. (4).

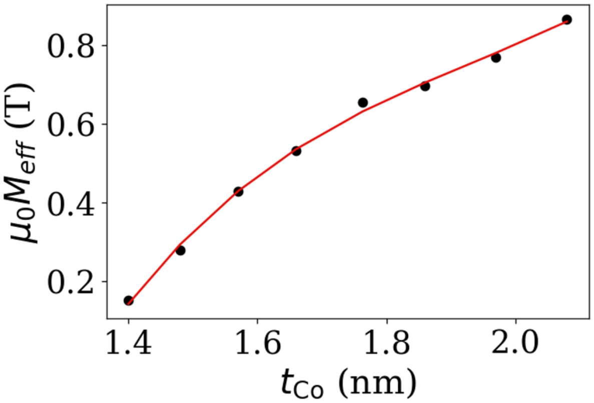

Figure S8: **Effective magnetization calibration.** Effective magnetization measured using conventional ST-FMR for samples with in-plane magnetic anisotropy.

REFERENCES AND NOTES

1. I. M. Miron, K. Garello, G. Gaudin, P.-J. Zermatten, M. V. Costache, S. Auffret, S. Bandiera, B. Rodmacq, A. Schuhl, P. Gambardella, Perpendicular switching of a single ferromagnetic layer induced by in-plane current injection. *Nature* **476**, 189–193 (2011).

2. L. Liu, C.-F. Pai, Y. Li, H. W. Tseng, D. C. Ralph, R. A. Buhrman, Spin-torque switching with the giant spin Hall effect of tantalum. *Science* **336**, 555–558 (2012).

3. U. H. Pi, K. Won Kim, J. Y. Bae, S. C. Lee, Y. J. Cho, K. S. Kim, S. Seo, Tilting of the spin orientation induced by Rashba effect in ferromagnetic metal layer. *Appl. Phys. Lett.* **97**, 162507 (2010).

4. L. Liu, O. J. Lee, T. J. Gudmundsen, D. C. Ralph, R. A. Buhrman, Current-induced switching of perpendicularly magnetized magnetic layers using spin torque from the spin Hall effect. *Phys. Rev. Lett.* **109**, 096602 (2012).

5. K. Garello, I. M. Miron, C. O. Avci, F. Freimuth, Y. Mokrousov, S. Blügel, S. Auffret, O. Boulle, G. Gaudin, P. Gambardella, Symmetry and magnitude of spin-orbit torques in ferromagnetic heterostructures. *Nat. Nanotechnol.* **8**, 587–593 (2013).

6. M. Hayashi, J. Kim, M. Yamanouchi, H. Ohno, Quantitative characterization of the spin-orbit torque using harmonic Hall voltage measurements. *Phys. Rev. B* **89**, 144425 (2014).

7. S. Woo, M. Mann, A. J. Tan, L. Caretta, G. S. D. Beach, Enhanced spin-orbit torques in Pt/Co/Ta heterostructures. *Appl. Phys. Lett.* **105**, 212404 (2014).

8. C.-F. Pai, M. Mann, A. J. Tan, G. S. D. Beach, Determination of spin torque efficiencies in heterostructures with perpendicular magnetic anisotropy. *Phys. Rev. B* **93**, 144409 (2016).

9. Y. Fan, X. Kou, P. Upadhyaya, Q. Shao, L. Pan, M. Lang, X. Che, J. Tang, M. Montazeri, K. Murata, L.-T. Chang, M. Akyol, G. Yu, T. Nie, K. L. Wong, J. Liu, Y. Wang, Y. Tserkovnyak, K. L. Wang, Electric-field control of spin-orbit torque in a magnetically doped topological insulator. *Nat. Nanotechnol.* **11**, 352–359 (2016).

10. C. O. Avci, A. Quindeau, C.-F. Pai, M. Mann, L. Caretta, A. S. Tang, M. C. Onbasli, C. A. Ross, G. S. D. Beach, Current-induced switching in a magnetic insulator. *Nat. Mater.* **16**, 309–314 (2017).

11. S.-H. C. Baek, V. P. Amin, Y.-W. Oh, G. Go, S.-J. Lee, G.-H. Lee, K.-J. Kim, M. D. Stiles, B.-G. Park, K.-J. Lee, Spin currents and spin-orbit torques in ferromagnetic trilayers. *Nat. Mater.* **17**, 509–513 (2018).

12. J. Mendil, M. Trassin, Q. Bu, M. Fiebig, P. Gambardella, Current-induced switching of YIG/Pt bilayers with in-plane magnetization due to Oersted fields. *Appl. Phys. Lett.* **114**, 172404 (2019).

13. M. Alghamdi, M. Lohmann, J. Li, P. R. Jothi, Q. Shao, M. Aldosary, T. Su, B. P. T. Fokwa, J. Shi, Highly efficient spin-orbit torque and switching of layered ferromagnet Fe_3GeTe_2. *Nano Lett.* **19**, 4400–4405 (2019).

14. X. Wang, J. Tang, X. Xia, C. He, J. Zhang, Y. Liu, C. Wan, C. Fang, C. Guo, W. Yang, Y. Guang, X. Zhang, H. Xu, J. Wei, M. Liao, X. Lu, J. Feng, X. Li, Y. Peng, H. Wei, R. Yang, D. Shi, X. Zhang, Z. Han, Z. Zhang, G. Zhang, G. Yu, X. Han, Current-driven magnetization switching in a van der Waals ferromagnet Fe_3GeTe_2. *Sci. Adv.* **5**, eaaw8904 (2019).

15. V. Gupta, T. M. Cham, G. M. Stiehl, A. Bose, J. A. Mittelstaedt, K. Kang, S. Jiang, K. F. Mak, J. Shan, R. A. Buhrman, D. C. Ralph, Manipulation of the van der Waals Magnet $Cr_2Ge_2Te_6$by spin–orbit torques. *Nano Lett.* **20**, 7482–7488 (2020).

16. W. Yanez, Y. Ou, R. Xiao, J. Koo, J. T. Held, S. Ghosh, J. Rable, T. Pillsbury, E. G. Delgado, K. Yang, J. Chamorro, A. J. Grutter, P. Quarterman, A. Richardella, A. Sengupta, T. McQueen, J. A. Borchers, K. A. Mkhoyan, B. Yan, N. Samarth, Spin and charge interconversion in dirac-semimetal thin films. *Phys. Rev. Appl.* **16**, 054031 (2021).

17. E. Cogulu, H. Zhang, N. N. Statuto, Y. Cheng, F. Yang, R. Cheng, A. D. Kent, Quantifying spin-orbit torques in antiferromagnet–heavy-metal heterostructures. *Phys. Rev. Lett.* **128**, 247204 (2022).

18. Y. Cheng, E. Cogulu, R. D. Resnick, J. J. Michel, N. N. Statuto, A. D. Kent, F. Yang, Third harmonic characterization of antiferromagnetic heterostructures. *Nat. Commun.* **13**, 3659 (2022).

19. J. Gibbons, T. Dohi, V. P. Amin, F. Xue, H. Ren, J.-W. Xu, H. Arava, S. Shim, H. Saglam, Y. Liu, J. E. Pearson, N. Mason, A. K. Petford-Long, P. M. Haney, M. D. Stiles, E. E. Fullerton, A. D. Kent, S. Fukami, A. Hoffmann, Large exotic spin torques in antiferromagnetic iron rhodium. *Phys. Rev. Appl.* **18**, 024075 (2022).

20. C. O. Avci, K. Garello, M. Gabureac, A. Ghosh, A. Fuhrer, S. F. Alvarado, P. Gambardella, Interplay of spin-orbit torque and thermoelectric effects in ferromagnet/normal-metal bilayers. *Phys. Rev. B* **90**, 224427 (2014).

21. N. Roschewsky, E. S. Walker, P. Gowtham, S. Muschinske, F. Hellman, S. R. Bank, S. Salahuddin, Spin-orbit torque and nernst effect in Bi-Sb/Co heterostructures. *Phys. Rev. B* **99**, 195103 (2019).

22. S. Karimeddiny, J. A. Mittelstaedt, R. A. Buhrman, D. C. Ralph, Transverse and longitudinal spin-torque ferromagnetic resonance for improved measurement of spin-orbit torque. *Phys. Rev. Appl.* **14**, 024024 (2020).

23. S. Karimeddiny, D. C. Ralph, Resolving discrepancies in spin-torque ferromagnetic resonance measurements: Lineshape versus linewidth analyses. *Phys. Rev. Appl.* **15**, 064017 (2021).

24. T. M. Cham, S. Karimeddiny, V. Gupta, J. A. Mittelstaedt, D. C. Ralph, Separation of artifacts from spin-torque ferromagnetic resonance measurements of spin-orbit torque for the low-symmetry van der Waals semi-metal $ZrTe_3$. *Adv. Quantum Technol.* **5**, 2100111 (2022).

25. F. Xue, C. Rohmann, J. Li, V. Amin, P. Haney, Unconventional spin-orbit torque in transition metal dichalcogenide–ferromagnet bilayers from first-principles calculations. *Phys. Rev. B* **102**, 014401 (2020).

26. F. Xue, P. M. Haney, Staggered spin hall conductivity. *Phys. Rev. B* **102**, 195146 (2020).

27. X. Fan, H. Celik, J. Wu, C. Ni, K.-J. Lee, V. O. Lorenz, J. Q. Xiao, Quantifying interface and bulk contributions to spin–orbit torque in magnetic bilayers. *Nat. Commun.* **5**, 3042 (2014).

28. X. Fan, A. R. Mellnik, W. Wang, N. Reynolds, T. Wang, H. Celik, V. O. Lorenz, D. C. Ralph, J. Q. Xiao, All-optical vector measurement of spin-orbit-induced torques using both polar and quadratic magneto-optic Kerr effects. *Appl. Phys. Lett.* **109**, 122406 (2016).

29. M. Montazeri, P. Upadhyaya, M. C. Onbasli, G. Yu, K. L. Wong, M. Lang, Y. Fan, X. Li, P. Khalili Amiri, R. N. Schwartz, C. A. Ross, K. L. Wang, Magneto-optical investigation of spin-orbit torques in metallic and insulating magnetic heterostructures. *Nat. Commun.* **6**, 8958 (2015).

30. Y. K. Kato, R. C. Myers, A. C. Gossard, D. D. Awschalom, Observation of the spin Hall effect in semiconductors. *Science* **306**, 1910–1913 (2004).

31. J. Lee, Z. Wang, H. Xie, K. F. Mak, J. Shan, Valley magnetoelectricity in single-layer MoS_2. *Nat. Mater.* **16**, 887–891 (2017).

32. D. C. Ralph, M. D. Stiles, Spin transfer torques. *J. Magn. Magn. Mater.* **320**, 1190–1216 (2008).

33. Y. Ou, C.-F. Pai, S. Shi, D. C. Ralph, R. A. Buhrman, Origin of fieldlike spin-orbit torques in heavy metal/ferromagnet/oxide thin film heterostructures. *Phys. Rev. B* **94**, 140414 (2016).

34. C.-F. Pai, Y. Ou, L. H. Vilela-Leao, D. C. Ralph, R. A. Buhrman, Dependence of the efficiency of spin hall torque on the transparency of Pt/ferromagnetic layer interfaces. *Phys. Rev. B* **92**, 064426 (2015).

35. S. Karimeddiny, T. M. Cham, D. C. Ralph, Y. K. Luo, Sagnac interferometry for high-sensitivity optical measurements of spin-orbit torque. arXiv:2109.13759 [cond-mat.mes-hall] (28 September 2021).

36. L. Zhu, K. Sobotkiewich, X. Ma, X. Li, D. C. Ralph, R. A. Buhrman, Strong damping-like spin-orbit torque and tunable Dzyaloshinskii–Moriya interaction generated by low-resistivity $Pd_{1-x}Pt_x$ alloys. *Adv. Funct. Mater.* **29**, 1805822 (2019).

37. L. Liu, T. Moriyama, D. C. Ralph, R. A. Buhrman, Spin-torque ferromagnetic resonance induced by the spin Hall effect. *Phys. Rev. Lett.* **106**, 036601 (2011).

38. S. Karimeddiny, “Spin-orbit torque metrology,” thesis, Cornell University, Ithaca, NY (2021).

39. J. Xia, P. T. Beyersdorf, M. M. Fejer, A. Kapitulnik, Modified Sagnac interferometer for high-sensitivity magneto-optic measurements at cryogenic temperatures. *Appl. Phys. Lett.* **89**, 062508 (2006).

40. A. Fried, M. Fejer, A. Kapitulnik, A scanning, all-fiber Sagnac interferometer for high resolution magneto-optic measurements at 820 nm. *Rev. Sci. Instrum.* **85**, 103707 (2014).

41. C. Kittel, On the theory of ferromagnetic resonance absorption. *Phys. Rev.* **73**, 155–161 (1948).
